# Alternative splicing in multiple myeloma is associated with the non-homologous end joining pathway

**DOI:** 10.1038/s41408-023-00783-0

**Published:** 2023-01-20

**Authors:** Enze Liu, Nathan Becker, Parvathi Sudha, Chuanpeng Dong, Yunlong Liu, Jonathan Keats, Gareth Morgan, Brian A. Walker

**Affiliations:** 1 Melvin and Bren Simon Comprehensive Cancer Center, Division of Hematology and Oncology, School of Medicine, Indiana University, Indianapolis, IN USA; 2 Center for Computational Biology and Bioinformatics, School of Medicine, Indiana University, Indianapolis, IN USA; 3 Department of Genetics, School of Medicine, Yale University, New Haven, CT USA; 4 Translational Genomics Research Institute (TGen), Integrated Cancer Genomics Division, Phoenix, AZ USA; 5 NYU Langone Medical Center, Perlmutter Cancer Center, NYU Langone Health, New York, NY USA

**Keywords:** Cancer genomics, Myeloma

## Abstract

Alternative splicing plays a pivotal role in tumorigenesis and proliferation. However, its pattern and pathogenic role has not been systematically analyzed in multiple myeloma or its subtypes. Alternative splicing profiles for 598 newly diagnosed myeloma patients with comprehensive genomic annotation identified primary translocations, 1q amplification, and *DIS3* events to have more differentially spliced events than those without. Splicing levels were correlated with expression of splicing factors. Moreover, the non-homologous end joining pathway was an independent factor that was highly associated with splicing frequency as well as an increased number of structural variants. We therefore identify an axis of high-risk disease encompassing expression of the non-homologous end joining pathway, increase structural variants, and increased alternative splicing that are linked together. This indicates a joint pathogenic role for DNA damage response and alternative RNA processing in myeloma.

## Introduction

Multiple myeloma (MM) is characterized by extensive inter-patient genomic heterogeneity due to various initiating events [1], and key genomic abnormalities driving the disease have been identified. Roughly half of patients carry primary translocations involving the immunoglobulin (Ig) loci, and can be divided into 5 main subtypes: t(11;14) (15%), t(4;14) (12%), t(14;16) (3%), t(14;20) (2%), and t(6;14) (1%), each of which results in over-expression of key oncogenes. The other half of patients have primary events involving gains of odd-numbered chromosomes, known as hyperdiploidy (57%). Secondary events cooperate with primary events to further facilitate disease progression. These secondary events include additional translocations, copy number variations, loss of heterozygosity, mutations, and epigenetic abnormalities [2]. *KRAS* (21%), *NRAS* (19%), *BRAF* (7%), *DIS3* (9%), *TENT5C* (6%), and *TP53* (3%) are amongst the most frequently mutated genes, some of which contribute to high-risk disease. Clinical factors including the International Staging System (ISS) [3], along with genomic factors such as gain or amplification 1q, del(17p), t(4;14), and ‘Double-Hit’ are often used as indicators for predicting poor prognosis [4, 5].

Although RNA expression has been utilized to identify high-risk patients [6, 7], other means of RNA processing have not, even though two genes involved in RNA processing are frequently mutated: *DIS3* and *TENT5C*. Alternative splicing (AS) is the selection of different combinations of splice sites within a pre-mRNA to generate variably spliced mRNAs, which further leads to various protein products [8]. AS is one of the fundamental forces that drive proteome diversity in humans [9], and is pervasive in cellular processes of malignancies, including proliferation, invasion, metastasis, and resistance to drugs [8, 10, 11]. However, AS is not reflected in overall gene expression levels.

Various studies have indicated the pathogenic roles of AS in MM [12–17]. AS-derived isoforms promote core pathways in MM, such as glycolysis [13] and the PI3K-Akt pathway [14, 15]. Some studies have illustrated the inhibitory effect of AS toward tumorigenesis when those isoforms are targeted in vivo, in vitro, and in clinical trials [16]. In acute myeloid leukemia (AML) [18], aberrant AS has been proven to be directly responsible for the resistance toward chimeric antigen receptor T (CAR-T) cell therapy. These efforts have indicated the oncogenic roles of AS in MM, and some abnormalities can be effectively targeted. Nonetheless, no study has utilized next-generation sequencing data in MM to systematically investigate AS abnormalities on a transcriptomic scale. Therefore, there is a need to identify AS driver events that can be further targeted and used as complementary approaches to existing treatment plans.

In this study, we analyzed newly diagnosed MM (NDMM) patient samples from the CoMMpass dataset and generated whole-transcriptome splicing profiles for 18 genomic subtypes. We systematically compared these profiles and identified similarities and differences in AS events at the gene and functional levels. Survival analysis was conducted on AS events and corresponding isoforms to measure their prognostic value. The key mechanisms associated with AS frequencies in NDMM were identified. In addition, we explored the potential regulators that control individual AS events. Our study unveils the complex and heterogenous nature of AS landscapes among all major subtypes in MM.

## Materials and methods

### Patient samples and annotations

RNA-seq data from the CoMMpass study was used to generate alternative splicing comparisons. From this dataset, 598 newly diagnosed MM patients with RNA-seq data were utilized. Samples were previously annotated for cytogenetic, copy number, mutation, and clinical data such as age [19]. RNA-seq from normal plasma cells (*n* = 5) was obtained from previously published data (GSE110486) [20]. Structural variation information was obtained from MMRF CoMMpass research portal IA16 from whole genome sequencing data.

### Data processing

Details of RNA-seq processing, alternative splicing analysis, and differential gene expression can be found in the Supplementary Methods and Supplementary Fig. 1.

### Pathway analysis

Differentially expressed and spliced genes were examined using GSEA [21] to identify dysregulated pathways. *P* < 0.05 was used as the threshold to filter out non-significant pathways.

### Survival analysis

Survival analysis using Cox regression was performed on all samples using the clinical annotations of CoMMpass IA16 to calculate progression free survival (PFS) and overall survival (OS) relating to AS events. Well-known high-risk factors were taken into account in multivariate analyses including the International Staging System (ISS), 1q gain or amplification, 17p deletion, t(14;16), t(4;14), *TP53* loss of function (mutation, deletion) and the previously defined Double-Hit [5]. Kaplan–Meier curves were plotted to display the survival probability and duration difference along with Logrank test.

### Correlation network construction

Undirected networks were constructed to measure the correlation between the splicing level of AS events and the expression 69 splicing factors (SFs) [22]. AS events and SFs were indicated as nodes while Spearman correlations between them were indicated by edges. Cytoscape was used to generate the network plots.

### Statistics analysis

Mann–Whitney U test was conducted for comparing the AS event frequency differences and number of structural variation differences. One-tailed t-test was used to compare the splicing level and expression level differences. Kolmogorov–Smirnov test was conducted for testing the normality of the distribution of number of structural variation events. Fisher’s exact test was to test if a genetic event is enriched in a group.

## Results

### Alternative splicing in multiple myeloma is driven by translocations, 1q amplification, and biallelic events in DIS3

**Fig. 1:**
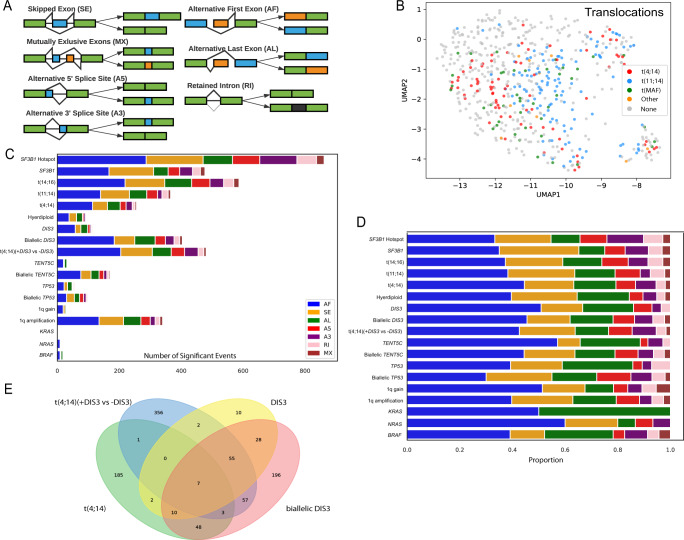
Alternative splicing types among different cytogenetic and mutation comparisons. **A** Seven fundamental AS types. **B** Unsupervised clustering of 598 NDMM samples by transcriptome splicing profiles with highlighted translocations. **C** Number of alternatively spliced events among genomic abnormality comparisons. **D** Composition of alternatively spliced events within each comparison. **E** Venn diagram of consistent AS events among t(4;14), t(4;14) + *DIS3*, biallelic *DIS3* and *DIS3* mutation comparisons.

Previously we determined that mutations in *SF3B1* resulted in increased AS in MM patient samples [12]. However, only 1.6% of patients have this mutation, indicating that AS may not be as important in MM than in other hematological malignancies, or that there are other mechanisms specific to MM that are at play. To determine the full extent of alternative splicing (Fig. 1A) in MM, we performed UMAP clustering of 598 NDMM samples using the top 1% most-variate events, Fig. 1B. A clear segregation could be observed between samples with or without IgH translocations (Fig. 1B). This is in line with other genomic studies where the primary translocation events drive clustering of gene expression and DNA methylation data [23–25].

To determine if other genomic factors could be involved in generating AS in MM we performed 18 comparisons that cover all major cytogenetic subgroups (IgH translocations and hyperdiploidy) and the most common mutations found in MM (*BRAF*, *KRAS*, *NRAS*, *DIS3*, *TENT5C*, and *TP53*), *SF3B1* mutation and *SF3B1* mutation hotspots, as well as those with biallelic abnormalities including *DIS3, TENT5C*, and *TP53*, chromosome 1q gain and amplification, plus one combination event where there is an association of *DIS3* mutations in the t(4;14) (t(4;14) + *DIS3* mutation). Sample size and comparison groups are described in Supplementary Table 1.

Differentially spliced events were identified in each comparison and further compared against a set of normal plasma cell RNA-seq data (*N* = 5) to remove any cell-type specific events. In total, 4422 AS events (|dPSI| >10%, TPM > 1, *P* < 0.05) were identified (Fig. 1C, Supplementary Tables 2, 3, and Supplementary Figs. 2, 3). Samples with *SF3B1* mutation hotspots contained the greatest number of AS events (*N* = 862), and samples with any *SF3B1* mutation had approximately half as many. Samples with primary *IGH* translocations had an equivalent number of AS events to those with *SF3B1* mutations, with t(14;16) having the most (*N* = 587) followed by t(11;14) (*N* = 366), and t(4;14) (*N* = 256), but very few events were shared by all three translocation groups (Supplementary Fig. 4). 1q amplification also resulted in a large number of AS events (*N* = 430) whereas 1q gain did not (*N* = 37) and the absolute splicing levels of events consistently increased from 1q gain to 1q amplification (Supplementary Fig. 4J). The proportion of AS event types was similar among all comparisons (Fig. 1D), with alternative first (AF) exons being the dominant event type, followed by skipped exons (SE), and alternative last exons (AL).

*DIS3* at 13q and *TENT5C* at 1p are frequently mutated in MM and play a part in RNA processing. Both genes are on chromosomal regions that are frequently deleted in MM patient samples and so we also looked at the effect of monoallelic and biallelic alterations at the loci. We identified increased AS in the biallelic state compared to the monoallelic state for both *DIS3* (*P* = 0.02) and *TENT5C* (*P* = 0.01), Fig. 1C. The same was true for *TP53*, albeit at lower levels. When the mutation, copy number loss, and bi-allelic AS events were compared there was a synergistic effect for *DIS3* and *TENT5C* biallelic status while an additive effect was observed for *TP53* (Supplementary Fig. 4B–D). This observation still held on corresponding gene levels (Supplementary Table 4).

As *DIS3* mutations were also enriched in the t(4;14) subgroup, we compared t(4;14) samples with and without *DIS3* mutations to determine the effect on AS. This analysis indicated an increased number of AS events as a result of *DIS3* mutations on a t(4;14) background (Fig. 1E). In addition, comparisons of biallelic *DIS3* with or without a t(4;14) compared against samples without either abnormality indicated an increase in AS events and the corresponding genes when both abnormalities are present (Fig. 1E, Supplementary Figs. 3, 4, Supplementary Table 4). As del(13q) is common in MM (~50% of patients) and is enriched in t(4;14) MM (95% with del(13q)), we compared samples with or without del(13q) which resulted in very few (*N* = 32) AS events, indicating that del(13q) is not a confounder in this comparison.

### Alternative splicing events affect key genes and pathways in multiple myeloma

**Fig. 2:**
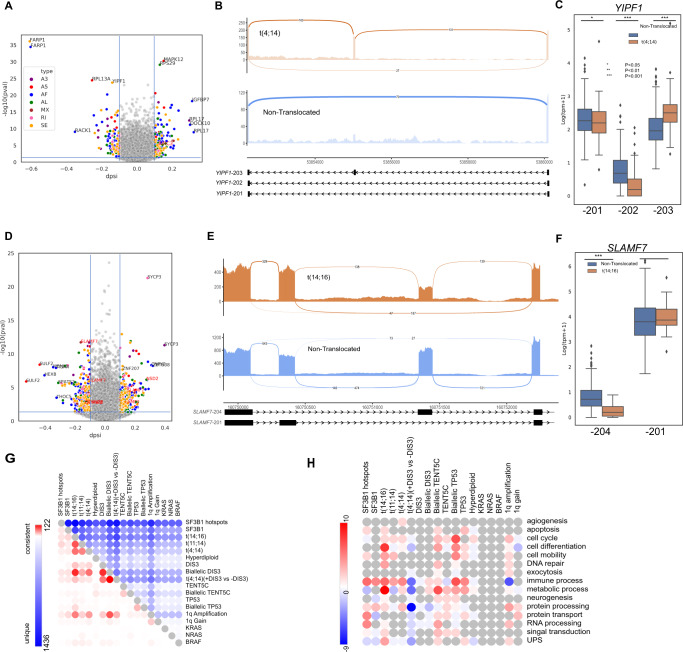
Heterogenous nature of AS among cytogenetic groups. **A**, **D** Volcano plots of AS events in t(4;14) and t(14;16), respectively. Genes labeled in red indicate documented oncogenes in MM. **B**, **E** Examples of AS events in each translocation subgroup including a skipped exon in *YIPF1* in t(4;14) and a mutually exclusive exon event in *SLAMF7* in t(14;16). **C**, **F** Expression level of transcripts involved in AS events in (**B**) and (**E**). **G** Consistent (red) and unique (blue) AS events among 18 cytogenetic comparisons. **H** Upregulated (red) and downregulated (blue) pathways derived from AS comparisons. *P < 0.05; **P < 0.01; ***P < 0.001.

We further examined the AS specific events within subgroups. In the t(4;14) comparison, 256 AS events were identified (Fig. 2A). These included *YIPF1*, a gene that encodes a protein and plays a role in the Golgi reassembly and glycan synthesis. An alternative transcript, *YIPF1*-203, was identified with an extra exon near the 3’ end compared to *YIPF1*-201 (canonical transcript) and *YIPF1*-202 with the exon skipped (Fig. 2B). As a result, *YIPF1*-203 was predicted to undergo nonsense-mediated decay (Supplementary Fig. 4G), while *YIPF1*-201 and *YIPF1*-202 were protein-coding transcripts [26]. Although expression of *YIPF1* at the gene level was not different, the *YIPF1*-203 transcript had significantly higher expression in the t(4;14) while the *YIPF1*-201 and *YIPF1*-202 transcripts had significantly higher expression in the non-translocated samples (Fig. 2C). Therefore, even though the overall gene expression level of *YIPF1* was similar, this gene may lose its function in the t(4;14) due to the high expression of the nonsense-mediated decay *YIPF1*-203 transcript.

In the t(14;16) comparison, *SLAMF7*, which encodes a cell surface immunotherapeutic antigen that can be targeted in MM [27] and immune cells [28], underwent a mutually exclusive exon event generating transcripts *SLAMF7-204* and *SLAMF7-201* (Fig. 2D, E). *SLAMF7-204* was expressed at significantly lower levels in t(14;16) samples (log_2_FC = −0.52, *P* = 3 × 10^−8^) while *SLAMF7-201* showed no significant expression difference between t(14;16) and non-translocated samples (Fig. 2F). Compared to the canonical *SLAMF7-203* transcript, *SLAMF7-201* contained different amino acid sequences at the intracellular domain regions (258–335) (Supplementary Fig. 4H) and was predicted to encode a transmembrane protein [29]. *SLAMF7-204* encoded an isoform without a transmembrane domain, and therefore was predicted to encode a secreted protein [29]. Cell surface bound *SLAMF7* is targeted by Elotuzumab therapy while secreted *SLAMF7* is used in diagnosis, monitoring, and assessment of response of such therapy [30]. Significantly less *SLAMF7*-204 would result in less secreted *SLAMF7*, and as a result, the response of Elotuzumab therapy may not be correctly assessed for t(14;16) patients.

### Alternative splicing events are highly unique among comparisons

When comparing the AS events identified in the different genomic comparisons, we found that most of them are unique with little overlap, except limited similarities within the translocation comparisons and within the *DIS3* comparisons (Fig. 2G).

Within the *DIS3*-related comparisons, mutation alone identified the fewest AS events (Fig. 1E), and other alterations built on top of these. For example, 88% of AS events in the *DIS3* mutation comparison were consistently identified in the biallelic *DIS3* comparison and 56% in the t(4;14) (+*DIS3* vs. −*DIS3*) comparison. In addition, the biallelic *D*IS3 ± t(4;14) comparisons identified significantly more additional unique AS events (Supplementary Fig. 4F). This may indicate that complete loss of wild-type *DIS3* has a profound effect on RNA splicing and that the co-occurrence of *DIS3* mutations in the t(4;14) may have a biological role in AS, perhaps through epigenetic coordination with *NSD2*.

Event-level heterogeneity also led to functional heterogeneity. Pathway analysis of AS genes indicated functional differences within the subgroup analysis (Fig. 2H and Supplementary Fig. 5). Common pathways can be found among translocation groups including metabolic process, immune process, and cell mobility, even though most of the categories are upregulated in t(14;16) and t(4;14) but inconsistently dysregulated in t(11;14). t(14;16) contributes the most number of dysregulated pathways with cell differentiation and metabolic process being the most upregulated.

*DIS3* abnormalities had a profound negative effect on many pathways with downregulation of genes. In particular, RNA processing pathways were downregulated across all *DIS3* categories. The combination of t(4;14) + *DIS3* dysregulated the most pathways, all of which were downregulated (in comparison to t(4;14) without *DIS3* mutation). Biallelic *DIS3* contributed the second most, which included immune and metabolic processes, RNA processing, and protein processing.

### Alternative splicing frequency is associated with the activity of the non-homologous end joining pathway

**Fig. 3:**
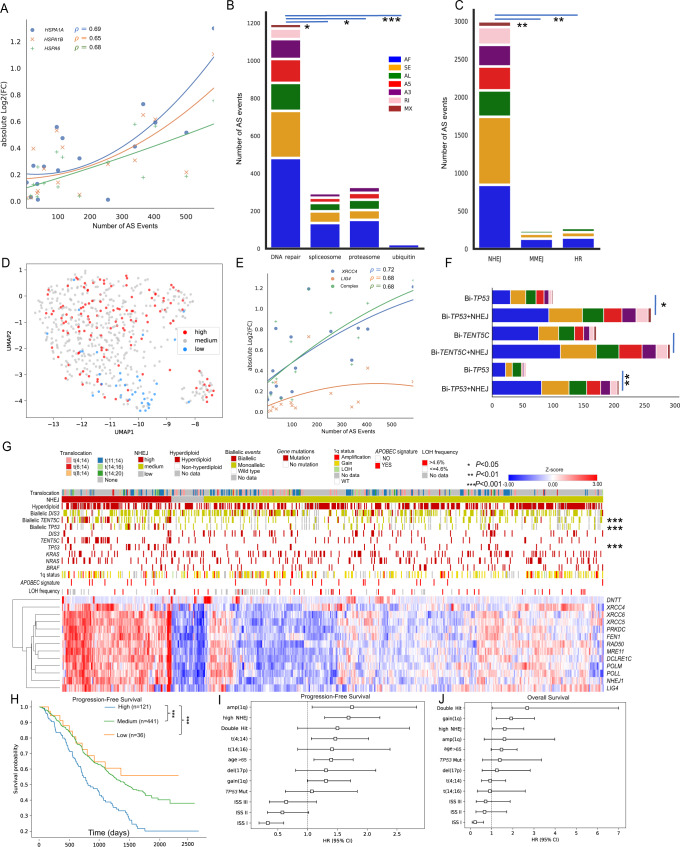
Non-homologous end joining (NHEJ) activity regulates AS frequency. **A** Spearman correlations between Log2-fold-change of heat shock protein genes and number of differentially spliced events in 16 comparisons. **B** Number of differentially spliced events between high and low activity groups of pathways. **C** Number of differentially spliced events between high and low activity groups of three DNA damage repair pathways. **D** Splicing patterns of samples in NHEJ-high and -low group across UMAP-clustered 598 NDMM samples. **E** Spearman correlations between expressions and splicing frequency on NHEJ genes *XRCC4*, *LIG4*, and *XRCC4*-DNA ligase IV complex. **F** Splicing frequency difference in: Biallelic *TP53* comparisons (Bi-*TP53*); Bi-*TP53* + NHEJ high activity subset comparisons; Biallelic *TENT5C* comparisons (Bi-*TENT5C*); Bi-*TENT5C* + NHEJ high activity comparisons. **G** Heatmaps of expression levels of NHEJ genes across 598 NDMM samples. **H** Kaplan–Meier curves indicating survival difference among NHEJ-high, -medium, and -low groups in PFS. **I**, **J** Multivariate survival analysis among 598 NDMM samples with NHEJ-high and other high-risk factors in PFS and OS. *P < 0.05; **P < 0.01; ***P < 0.001.

We speculated that spliceosome expression regulates the splicing frequency of transcripts. For each genetic comparison, we calculated the absolute log_2_(Fold-Change) of all spliceosome genes and performed unsupervised clustering (Supplementary Fig. 6). Four heat shock proteins, *HSPA1L*, *HSPA1A*, *HSPA1B*, and *HSPA6*, were clustered together, of which three correlated highly with splicing frequency (*ρ* > 0.65, Spearman correlation, Fig. 3A). All three genes encode proteins that belong to the Hsp70 protein family that contains various protein homologs, including Hsp73, Hsp70B’ and Hsp72 [31]. Hsp73 is the constitutive form and is encoded by the spliceosome gene *HSPA8* [31]. Hsp72 (encoded by *HSPA1A* and *HSPA1B*) and Hsp70B’ (encoded by *HSPA6*) are induced when the cell is under stress [31]. The Hsp70 protein family is a key part in maintaining protein homeostasis and cell survival [32]. Moreover, in the spliceosome, the three homologs act as part of the Prp19 complex (Prp19C) [33, 34] which is involved in multiple cellular processes, including splicing, transcription elongation, DNA repair, and protein degradation.

To determine the effect of these pathways, we empirically defined high and low-expression groups based on curated KEGG pathway gene lists [34, 35] (Supplementary Fig. 7) and conducted differential splicing analysis between the two groups. Among the five pathways, DNA repair reflected significantly more (*P* < 0.05, one-sided Mann–Whitney U test) AS events (*N* = 1197) than other pathways (Fig. 3B), and also more than any of the cytogenetic groups that were previously defined. We further determined the importance of the DNA repair pathway by investigating the three main mechanisms responsible: homologous recombination (HR), non-homologous end joining (NHEJ) and microhomology-mediated end joining (MMEJ) [36]. The NHEJ pathway comparison (Supplementary Fig. 7E) identified significantly more (*P* < 0.05, one-sided Mann–Whitney U test) AS events (*N* = 2999) than the other two DNA repair pathway comparisons (Fig. 3C). Moreover, samples in the low NHEJ expression group were clustered together (Fig. 3D).

Among the 13 genes in the NHEJ pathway, 12 showed a significant expression difference between the high and low groups with up to a 4-fold change in expression (Supplementary Fig. 8). *XRCC4* and *LIG4*, which together encode the DNA ligase IV complex that is essential for NHEJ [37], had the highest fold-change in expression and correlation with splicing frequencies (*ρ* = 0.72 and *ρ* = 0.68 Spearman correlation, Fig. 3E).

Samples in the NHEJ-high expression group were enriched for mutations in *TP53* (15% vs. 6%, *P* = 2 × 10^−5^, Fisher’s exact test), biallelic *TENT5C* (21% vs. 10%, *P* = 1 × 10^−9^) and biallelic *TP53* (10% vs. 4%, *P* = 3 × 10^−5^) events (Fig. 3F, G). The combination of these abnormalities with high expression of the NHEJ pathway resulted in significantly more differential AS events than the abnormality alone, for example, biallelic *TP53* + high NHEJ compared against samples without biallelic *TP53* abnormalities results in more differential AS events than biallelic *TP53* alone (Fig. 3F). The NHEJ-high expression group was not associated with other DNA repair abnormalities such as APOBEC signatures [19] and high loss of heterozygosity (LOH) frequency (>4.6%) (Fig. 3G) [38]. However, the NHEJ-high group was associated with more structural variants (mean 87 vs. 19, respectively, one-sided Mann–Whitney U-test *P* = 6 × 10^−4^) and a worse outcome compared to the others for PFS (Logrank test statistic = 7.3, *P* = 0.007), Fig. 3H, which remained significant in a multivariate analysis with other well-known risk factors for PFS (HR = 1.7, CI = (1.3,2.2), *P* = 1.5 × 10^–4^) and for OS (HR = 1.6, CI = (1.0,2.5), *P* = 0.03), including 1q gain/amp, 17p deletion, high-risk translocations, *TP53* mutation, and the previously defined Double-Hit, Fig. 3I, J [5].

Here we bring together the association of DNA instability, an increased number of SVs, enrichment of abnormalities in *TP53* and *TENT5C*, increased activity in the NHEJ pathway, which in turn affects spliceosome component expression and AS of RNA transcripts.

### Alternative splicing events are associated with outcome within cytogenetic subgroups

We subsequently conducted a survival analysis and evaluated the effect on outcome associated with each AS event both within their genomic subgroup and among all NDMM samples, Supplementary Table 5. In total, 1624 events showed a significant association with survival for either PFS or OS. Among them, the t(4;14) + *DIS3* comparison offered the highest number (*N* = 481), followed by t(11;14) (*N* = 218) and t(4;14) (*N* = 120) in univariate analysis for PFS while *DIS3* mutation reflected the highest number (*N* = 114) in multivariate analysis in PFS. Biallelic *TP53* (*N* = 100, 100%) and *TP53* mutation (*N* = 56, 100%) reflected the highest number of significant events in univariate analysis in OS and the highest proportion in PFS.

**Fig. 4:**
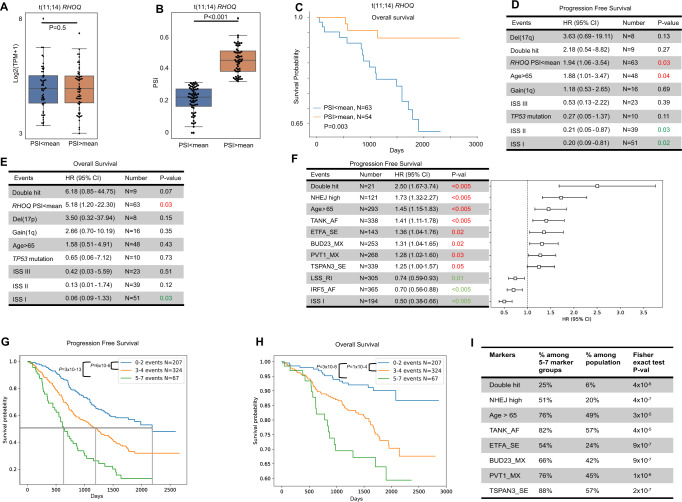
Association of AS events and outcome. **A** Gene level expression of *RHOQ* in the two AS groups is the same in t(11;14) samples. **B** Splicing of the AF event in *RHOQ* in the two t(11;14) groups. **C** *RHOQ* splicing levels are associated with OS in t(11;14) patients. **D**, **E** Multivariate analysis of t(11;14) patients showing that the association of AS of *RHOQ* with poor PFS and OS is independent of other high-risk factors. **F** Predicted prognostic model of the 200 AS events with most variation across samples with well-known high-risk factors identifies 7 AS events associated with PFS. **G**, **H** Kaplan–Meier curves indicating the additive effects of samples carrying various number of the 10 poor prognostic markers defined in F for PFS and OS. *P*-values from the Logrank test and median PFS are shown. **I** Percentage of poor prognostic markers within the high-risk group compared against the entire population.

Examples of these events included an AF event in *RHOQ* within the t(11;14) comparison. *RHOQ* encodes a GTPase that binds to a variety of effector proteins to regulate cellular responses. The mean PSI of this event was used to divide all t(11;14) samples into two subgroups, and although there was no gene level expression difference between the two t(11;14) groups (Fig. 4A, B), the ‘PSI < mean’ group was associated with a significantly worse OS compared to the ‘PSI > mean’ group (Fig. 4C). Furthermore, ‘PSI < mean’ maintained its association with high-risk in a multivariate analysis for PFS (Fig. 4D) and OS (Fig. 4E), suggesting that this AF event can independently define a high-risk subgroup in t(11;14) patients.

### Alternative splicing events are associated with high-risk multiple myeloma

We further investigated the independent prognostic effect of these events in a multivariate model with existing high-risk markers across all newly diagnosed patient samples. The top 200 expressed AS events with the highest variation across the entire population were selected and the mean PSI was used to divide the population into ‘<mean’ and ‘>mean’ groups. A backward elimination approach was used to select the predictors of poor PFS using Cox regression and as a result the final model contained 11 predictors in which 7 of them were AS markers (Fig. 4F) and reflected a high overall prediction performance (C-index = 0.68).

Among all AS markers, five were associated with unfavorable prognosis while two were indicators of favorable prognosis. We have previously shown that accumulation of poor prognosis markers results in a reduced outcome and so for the eight markers associated with unfavorable prognosis (five AS markers, NHEJ-high, Double-Hit and age ≥ 65), we tested whether patients with an increasing number of markers would result in decreased outcome. The samples were divided into three subgroups based on the number of poor prognosis markers they carried: low risk (0–2 markers), medium risk (3–4 markers), and high risk (5–7 markers) groups (no samples contained more than 7 markers). As the samples contained more markers, an additive risk was observed with the high-risk group (*N* = 67) reflecting a significantly worse median PFS and OS compared to the low-risk group (*N* = 207) (PFS median = 630 vs. 2185 days, *P* = 3 × 10^−13^, Logrank test, Fig. 4G; OS median not reached, *P* = 3 × 10^−6^, Fig. 4H). The high-risk group constituted ~11% of the entire population and were significantly enriched for the eight unfavorable markers (Fig. 4I). Sashimi plots and box plots for these high-risk AS events can be found in Supplementary Fig. 9, but included several genes implicated in MM biology, including *TANK* and *PVT1*.

### AS levels are highly correlated with expression of splicing factors

**Fig. 5:**
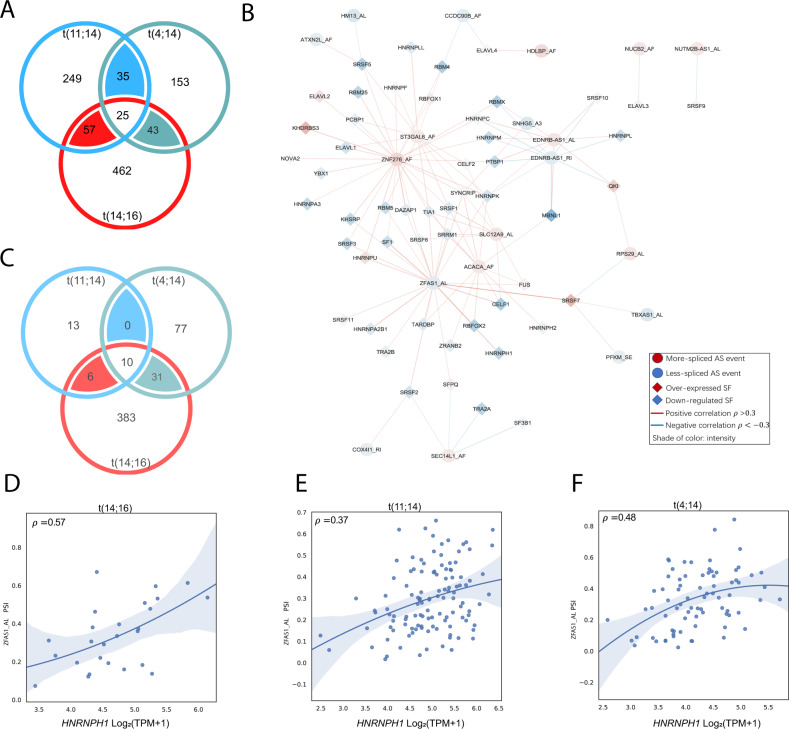
Activity of splicing factors (SF) is associated with the alternative splicing (AS) levels among translocations. **A** Venn diagrams indicating the consistent events among three translocations. **B** SF-AS correlation network among t(4;14) samples and consistent high-quality events among translocations. Spearman correlations were indicated by edges while log-fold-change of expression levels of SFs and *dPSI* of AS events were indicated by node. **C** Venn diagrams indicating the consistent high SF-AS correlations (>0.3) among 25 consistent events from (**A**). **D**–**F** An example SF-AS correlation consistently across translocations.

To understand the potential roles of splicing factors in regulating AS in MM we systematically studied the association between the expression of 69 splicing factors [22] and the splicing levels of 25 AS events identified in all three translocation subgroups (Fig. 5A). An empirical threshold (|*ρ*| > 0.3, Spearman correlation) was set to preserve high correlations, from which three correlation networks were constructed: t(11;14) Fig. 5B, t(14;16), and t(4;14) (Supplementary Fig. 10). Edge and node values of overlapped correlation networks can be found in Supplementary Table 6. Among the three networks, most events were regulated by the combinational effects from the up- or downregulation of splicing factors, while very few events were only associated with a single splicing factor. A large number of pairwise-consistent correlations existed (Fig. 5C) with 55% of correlations in t(11;14) and 35% in t(4;14) samples being consistent with other comparisons. Ten correlations were found to consistently exist among all three networks, eight of which are with an AL event in *ZFAS1*, whose splicing level was consistently lower in the three translocation comparisons compared to non-translocated samples. For example, the correlation between AL_*ZFAS1* splicing and *HNRNPH1* expression remained moderately high in all three translocations (Fig. 5D–F) as well as in hyperdiploid comparisons.

## Discussion

We and others have shown that the primary genetic events in myeloma, namely *IGH* translocations and hyperdiploidy, determine the trajectory of the cell with respect to gene expression, DNA methylation, mutational landscapes, and structural abnormalities [19, 39, 40]. Here, we show that this is also true of alternative RNA splicing where the translocation and hyperdiploid subgroups drive the patterns seen. We have previously shown that mutations in *SF3B1* can result in increased alternative splicing and here we extend that analysis to show that translocations, copy number abnormalities, and mutations in *DIS3* and *TENT5C* contribute significantly to alternative splicing, especially when both alleles are affected through deletion or mutation.

Moreover, here we show that this is exemplified by alterations in the NHEJ pathway where increased expression of pathway genes has been previously associated with poor outcome, and here is associated with increased alternative splicing. The group with high NHEJ expression were enriched for *TP53* and *TENT5C* abnormalities, highlighting the association between NHEJ and alternative splicing that has been documented in other diseases [41].

DNA damage repair has been linked to alternative splicing by two mechanisms [41]. First, heat shock protein 70 s (Hsp70s) are molecular chaperones that act as not only a fundamental component of spliceosome, but chaperone proteins that are recruited as cellular response toward DNA damage [42–44]. Second, DNA damage response can regulate splicing factors through expression changes, post-translational modifications, and cellular re-localization [41, 45]. *SRSF10* and *SRSF6* have been shown to connect these two mechanisms in vitro [46, 47], and we provided evidence to indicate the existence of both mechanisms in MM. Hence, it is no surprise to see the deterministic role of NHEJ in shaping the alternative splicing landscape in NDMM. We detected ~5-fold more structural variants in the samples with high expression of NHEJ genes, and speculate that these are more genomically unstable, resulting in more SVs that trigger a highly upregulated NHEJ pathway activity, which further leads to more aberrant AS events. Taken together, we consider SV-NHEJ-AS as an interacting network, in which each component may potentially serve as a biomarker of high-risk disease or novel molecular vulnerability.

*DIS3* abnormalities were also associated with higher levels of AS, especially when biallelic abnormalities affected *DIS3*. Although *DIS3* is a known component of the RNA exosome complex, it has been shown to regulate splicing factor expression in yeast [48], This effect may account for the increased AS in this group, however, *DIS3* has also been shown to impact genomic stability in human B cells and yeast, and so there may also be a link with DNA damage repair in these samples [49, 50]. *DIS3* mutations are enriched in t(4;14) patients [19], so we also examined the combination of these events and identified an increase in AS events when both abnormalities were present. The t(4;14) dysregulates *NSD2*, a histone methyltransferase, affecting chromatin structure and function. In AML there is evidence of a coordinated alterations in *IDH2* (epigenetic regulator) and *SRSF2* (splicing factor), resulting in distinct splicing changes and increased stalling of RNA polymerase II [51]. A similar phenomenon in MM may exist with *NSD2* and *DIS3*, and indeed the splicing landscape presented here aligns with previous reports of alternative active promoters with altered chromatin marks in MM [52].

Splicing factors are fundamental components of the spliceosome that control the splicing process and small molecules have been developed to target them in diseases with aberrant splicing [53]. We have shown that in MM subtypes, some events were highly correlated with the activity of a few splicing factors and such correlations can exist among various subtypes (e.g., *HNRNPH1* among three translocation groups), making these factors potential common targets.

Collectively, our study not only revealed the AS landscapes and their functional impact, but also their associated risks and clinical impact. The driving force of AS frequency in NDMM was also unveiled. This brought novel understanding of how AS is regulated and involved as part of the MM machinery, but also novel events and pathways that can be potentially targeted.

## Supplementary information

Supplementary Methods

Supplementary Figures

Supplementary Tables

## Data Availability

All sequencing datasets have been previously published and can be accessed through dbGAP Study Accession number phs000748 (MMRF CoMMpass dataset) and GEO GSE110486 (normal plasma cell dataset).
