# Supplementary material for: Alternative splicing in multiple myeloma is associated with the non-homologous end joining pathway
Source: Blood Cancer J. 2023 Jan 20;13(1):16. doi: 10.1038/s41408-023-00783-0 (PMC9859791; doi:10.1038/s41408-023-00783-0)
Supplement: Supplementary file 1 — Supplementary Methods [file 41408_2023_783_MOESM1_ESM.pdf]

Title:

Alternative Splicing in Multiple Myeloma is Associated with the Non-Homologous End Joining Pathway

Authors:

Enze Liu[1], Nathan Becker[1], Parvathi Sudha[1], Chuanpeng Dong[2,3], Yunlong Liu[2], Jonathan Keats[4], Gareth Morgan[5], Brian A. Walker[1,2]

Affiliations:

[1]Melvin and Bren Simon Comprehensive Cancer Center, Division of Hematology and Oncology, School of Medicine, Indiana University, Indianapolis, IN, USA

[2]Center for Computational Biology and Bioinformatics, School of Medicine, Indiana University, Indianapolis, IN, USA

[3]Department of Genetics, School of Medicine, Yale University, New Haven, CT, USA

[4]Translational Genomics Research Institute (TGen), Integrated Cancer Genomics Division, Phoenix, AZ, USA

[5]NYU Langone Medical Center, Perlmutter Cancer Center, NYU Langone Health, New York, NY, USA

Correspondence: Brian A. Walker, C310 Walther Hall, 980 W Walnut St, Indiana University, Indianapolis, IN, 46202. bw75@iu.edu

Supplementary Methods

RNA-seq Data Processing

>60 million read-pairs were sequenced per sample. Reads were evaluated by Fastqc (1) and low-quality reads and adapters identified and removed using Trimmomatic (2). Passing reads were aligned by STAR (3) with HG38 genome and Gencode V35 (hg38) genomic annotation using 'two-pass' mode. Transcriptome was quantified by Salmon (4) (Quasi-mapping mode) using Hg38 reference Genrome (a combined reference of Hg38 reference genome, Gencode V35 transcriptome and decoy sequences) with the following parameters (K-mer=31, standard EM algorithm and 'ValidateMappings'). Transcript-level and gene-level expression was measured in transcript-per-million (TPM).

Alternative Splicing Analysis

To identify differentially spliced events, groups were compared using SUPPA2 (5), which takes the transcript-level expression profile (generated from Salmon) and genomic annotation to calculate the 'Percentage of Spliced-In' (PSI) for each sample and the average splicing difference (ΔPSI ($dPSI$)) between the two groups for each event (6). PSI measures the splicing level of individual AS event with 0 being completely spliced and 1 being completely retained. Seven types of AS events were called (**Figure 1A**). High quality differential spliced events were defined using TPM >1, *P*<0.05 from independent (paired if two groups were from paired samples) T-test, >50% samples in either group with detected junction reads ($PSI \neq 1$), and $|dPSI| >10\%$. Significant events that were differentially spliced (P<0.05, independent t-test and $|dPSI| >10\%$) compared to normal BMPCs were kept (**Supplementary Figure 1**).

UMAP plots

SUPPA2 detects differential splicing by the expression ratio of transcript variants involved in each AS event. We similarly used this ratio of expressed transcript variants (TPM>1) involved in the top 1% most variable AS events across all samples as features to generate UMAP plots.

Differential Gene Expression

Log_2(TPM+1) transformation was applied to remove missing values and rescale expression levels. Limma (7) was subsequently applied to identify differentially expressed genes and estimate their fold changes, in which a moderated t-test was performed to normalize bias among samples and correct the differential expression. Transcript-level differential expression was performed in the same way. Differentially expressed genes had a false discovery rate (FDR)<0.05, absolute log_2-fold-change >0.48 (fold change >1.4 or <0.71), log_2(TPM+1)>1 in comparisons.

Pathway analysis

GSEA(8) was used to perform pathway analysis for differentially expressed or spliced genes. 'Gene Ontology Biological Process'(9) was used as the pathway set.

Defined high and low activity group based on pathway activity

For the 'ubiquitin', 'proteasome', 'spliceosome', 'homologous recombination' and 'non-homologous end joining' pathways, the gene set from KEGG was used. For the 'DNA repair' pathway, the KEGG definition contained more than 500 genes so a signature of 17 genes defined for MM were used (10). For the 'microhomology-mediated end joining (MMEJ)' pathway, no KEGG definition existed and so a previously published signature of

6 genes was used (11). Unsupervised hierarchical clustering was conducted for each of the pathways across all samples based on the expression level of genes in the set (**Supplementary Figure 8**). A complete list of differential pathway samples can be found in **Supplementary Table 6**.

APOBEC annotation

APOBEC signature information on samples was taken from a previous publication (Walker et al. Identification of novel mutational drivers reveals oncogene dependencies in multiple myeloma, Blood 2018). In this respect, the dominant APOBEC signature was utilized usually consisting of >30% APOBEC related mutations in the sample.

References

1. Andrews S. FastQC: a quality control tool for high throughput sequence data. Babraham Bioinformatics, Babraham Institute, Cambridge, United Kingdom; 2010.
2. Bolger AM, Lohse M, Usadel B. Trimmomatic: a flexible trimmer for Illumina sequence data. Bioinformatics. 2014;30(15):2114-20.
3. Dobin A, Davis CA, Schlesinger F, Drenkow J, Zaleski C, Jha S, et al. STAR: ultrafast universal RNA-seq aligner. Bioinformatics. 2013;29(1):15-21.
4. Patro R, Duggal G, Love MI, Irizarry RA, Kingsford C. Salmon provides fast and bias-aware quantification of transcript expression. Nature methods. 2017;14(4):417-9.
5. Trincado JL, Entizne JC, Hysenaj G, Singh B, Skalic M, Elliott DJ, et al. SUPPA2: fast, accurate, and uncertainty-aware differential splicing analysis across multiple conditions. Genome biology. 2018;19(1):1-11.
6. Schafer S, Miao K, Benson CC, Heinig M, Cook SA, Hubner N. Alternative Splicing Signatures in RNA-seq Data: Percent Spliced in (PSI). Curr Protoc Hum Genet. 2015;87:11 6 1- 6 4.
7. Diboun I, Wernisch L, Orengo CA, Koltzenburg M. Microarray analysis after RNA amplification can detect pronounced differences in gene expression using limma. BMC Genomics. 2006;7:252.
8. Subramanian A, Tamayo P, Mootha VK, Mukherjee S, Ebert BL, Gillette MA, et al. Gene set enrichment analysis: a knowledge-based approach for interpreting genome-wide expression profiles. Proceedings of the National Academy of Sciences. 2005;102(43):15545-50.
9. Ashburner M, Ball CA, Blake JA, Botstein D, Butler H, Cherry JM, et al. Gene ontology: tool for the unification of biology. 2000;25(1):25-9.
10. Kassambara A, Gourzones-Dmitriev C, Sahota S, Rème T, Moreaux J, Goldschmidt H, et al. A DNA repair pathway score predicts survival in human multiple myeloma: the potential for therapeutic strategy. Oncotarget. 2014;5(9):2487.
11. Sharma S, Javadekar SM, Pandey M, Srivastava M, Kumari R, Raghavan SC. Homology and enzymatic requirements of microhomology-dependent alternative end joining. Cell death & disease. 2015;6(3):e1697-e.
