# Supplementary material for: Alternative splicing in multiple myeloma is associated with the non-homologous end joining pathway
Source: Blood Cancer J. 2023 Jan 20;13(1):16. doi: 10.1038/s41408-023-00783-0 (PMC9859791; doi:10.1038/s41408-023-00783-0)
Supplement: Supplementary file 2 — Supplementary Figures [file 41408_2023_783_MOESM2_ESM.pdf]

Title:

Alternative Splicing in Multiple Myeloma is Associated with the Non-Homologous End Joining Pathway

Authors:

Enze Liu[1], Nathan Becker[1], Parvathi Sudha[1], Chuanpeng Dong[2,3], Yunlong Liu[2], Jonathan Keats[4], Gareth Morgan[5], Brian A. Walker[1,2]

Affiliations:

[1]Melvin and Bren Simon Comprehensive Cancer Center, Division of Hematology and Oncology, School of Medicine, Indiana University, Indianapolis, IN, USA

[2]Center for Computational Biology and Bioinformatics, School of Medicine, Indiana University, Indianapolis, IN, USA

[3]Department of Genetics, School of Medicine, Yale University, New Haven, CT, USA

[4]Translational Genomics Research Institute (TGen), Integrated Cancer Genomics Division, Phoenix, AZ, USA

[5]NYU Langone Medical Center, Perlmutter Cancer Center, NYU Langone Health, New York, NY, USA

Correspondence: Brian A. Walker, C310 Walther Hall, 980 W Walnut St, Indiana University, Indianapolis, IN, 46202. bw75@iu.edu

Supplementary Methods

RNA-seq Data Processing

>60 million read-pairs were sequenced per sample. Reads were evaluated by Fastqc (1) and low-quality reads and adapters identified and removed using Trimmomatic (2). Passing reads were aligned by STAR (3) with HG38 genome and Gencode V35 (hg38) genomic annotation using 'two-pass' mode. Transcriptome was quantified by Salmon (4) (Quasi-mapping mode) using Hg38 reference Genrome (a combined reference of Hg38 reference genome, Gencode V35 transcriptome and decoy sequences) with the following parameters (K-mer=31, standard EM algorithm and 'ValidateMappings'). Transcript-level and gene-level expression was measured in transcript-per-million (TPM).

Alternative Splicing Analysis

To identify differentially spliced events, groups were compared using SUPPA2 (5), which takes the transcript-level expression profile (generated from Salmon) and genomic annotation to calculate the 'Percentage of Spliced-In' (PSI) for each sample and the average splicing difference (ΔPSI ($dPSI$)) between the two groups for each event (6). PSI measures the splicing level of individual AS event with 0 being completely spliced and 1 being completely retained. Seven types of AS events were called (**Figure 1A**). High quality differential spliced events were defined using TPM >1, *P*<0.05 from independent (paired if two groups were from paired samples) T-test, >50% samples in either group with detected junction reads ($PSI \neq$1), and $|dPSI| >$10%. Significant events that were differentially spliced (P<0.05, independent t-test and $|dPSI| >$10%) compared to normal BMPCs were kept (**Supplementary Figure 1**).

UMAP plots

SUPPA2 detects differential splicing by the expression ratio of transcript variants involved in each AS event. We similarly used this ratio of expressed transcript variants (TPM>1) involved in the top 1% most variable AS events across all samples as features to generate UMAP plots.

Differential Gene Expression

Log_2(TPM+1) transformation was applied to remove missing values and rescale expression levels. Limma (7) was subsequently applied to identify differentially expressed genes and estimate their fold changes, in which a moderated t-test was performed to normalize bias among samples and correct the differential expression. Transcript-level differential expression was performed in the same way. Differentially expressed genes had a false discovery rate (FDR)<0.05, absolute log_2-fold-change >0.48 (fold change >1.4 or <0.71), log_2(TPM+1)>1 in comparisons.

Defined high and low activity group based on pathway activity

For the 'ubiquitin', 'proteasome', 'spliceosome', 'homologous recombination' and 'non-homologous end joining' pathways, the gene set from KEGG was used. For the 'DNA repair' pathway, the KEGG definition contained more than 500 genes so a signature of 17 genes defined for MM were used (8). For the 'microhomology-mediated end joining (MMEJ)' pathway, no KEGG definition existed and so a previously published signature of 6 genes was used (9). Unsupervised hierarchical clustering was conducted for each of the pathways across all samples based on the expression level of genes in the set (**Supplementary Figure 7**). A complete list of differential pathway samples can be found in **Supplementary Table 7**.

Supplementary Figures

Supplementary Figure 1. Workflow of this study

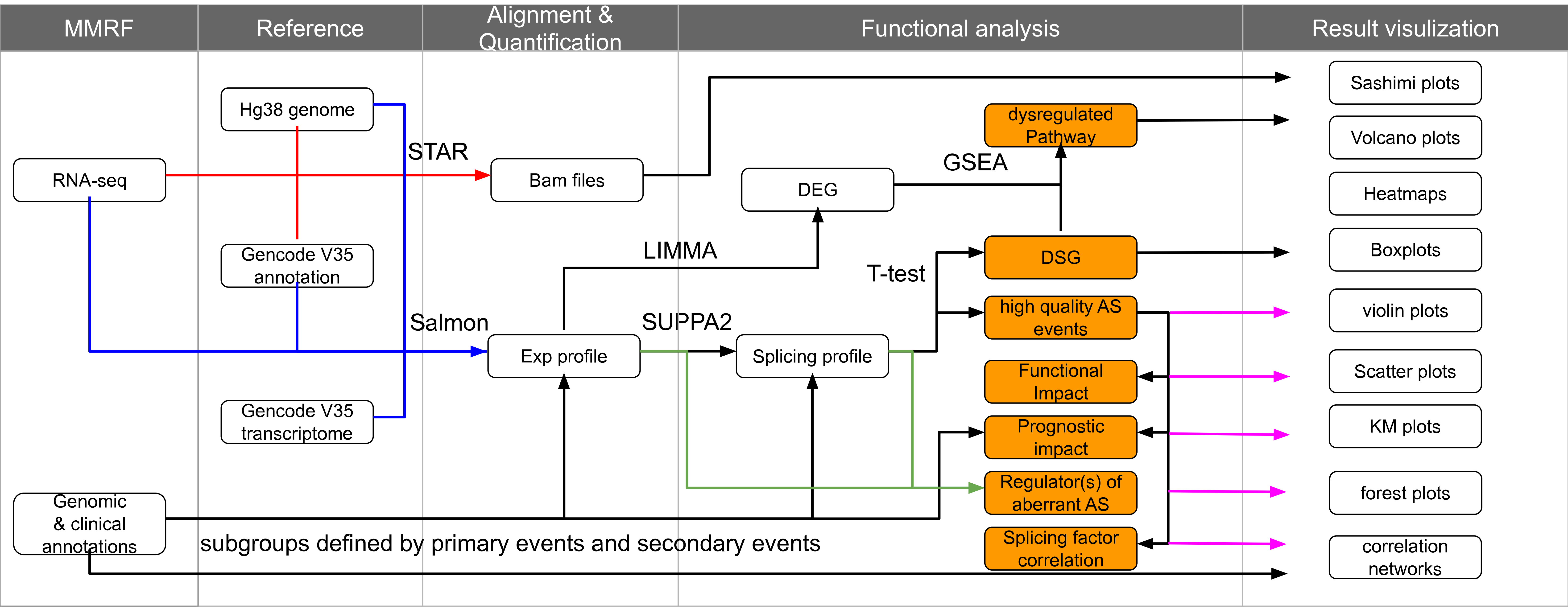

Supplementary Figure 2. Number of AS events identified from 18 comparisons.
(A) Number of AS events with a positive dPSI in the treatment group of each comparison.
(B) Number of AS events with a negative dPSI in the treatment group of each comparison.

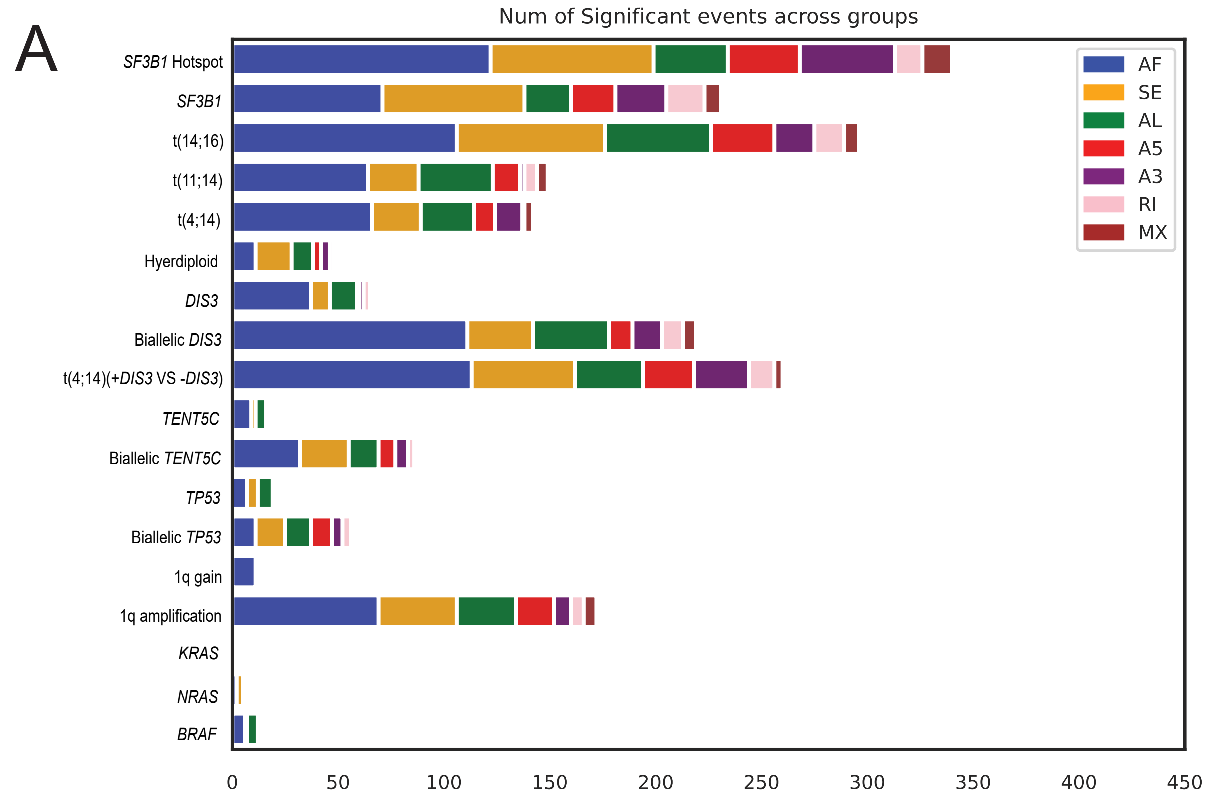

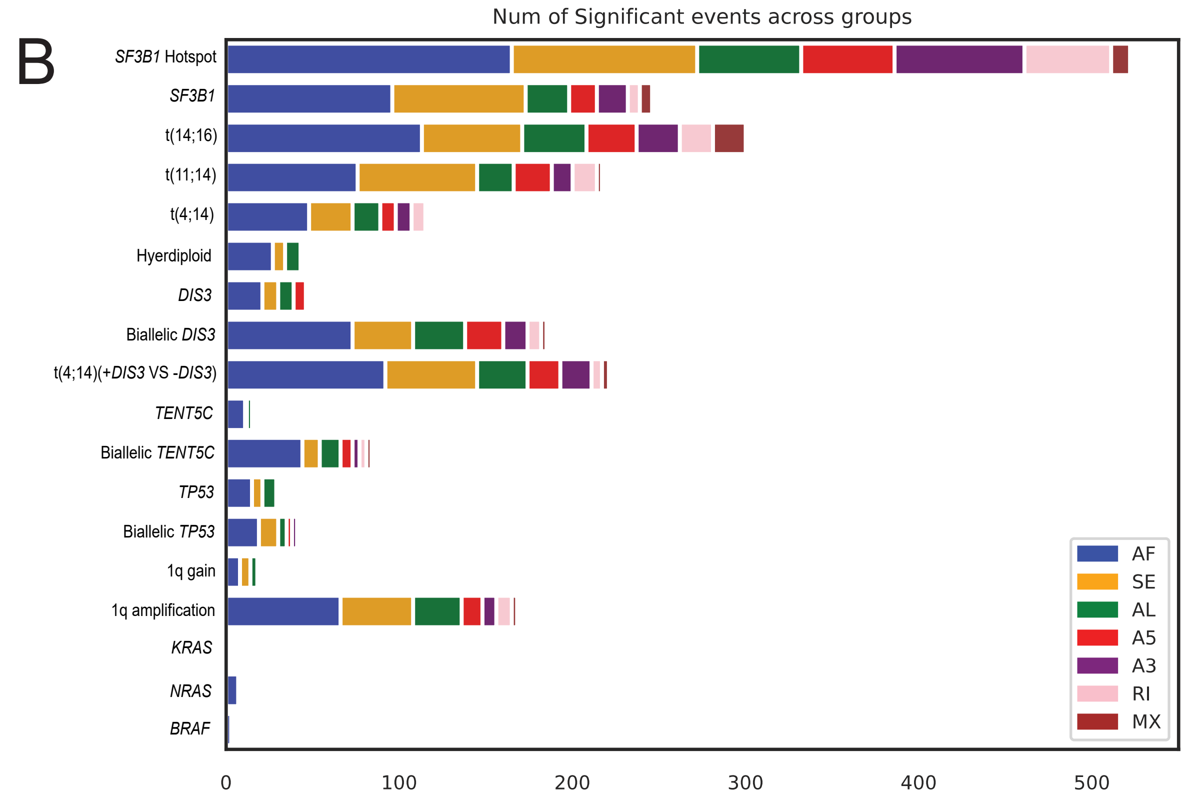

Supplementary Figure 3. Volcano plots of identified AS events in: (A) Biallelic DIS3 comparison; (B) DIS3 mutation comparison; (C) Biallelic TENT5C comparison; (D) SF3B1 mutation comparison; (E) SF3B1 mutation hotspot comparison; (F) t(4;14)+DIS3 comparison; (G) 1q amplification comparison; (H) t(11;14) comparison. (I) DIS3+t(4;14) vs no_t(4;14)+DIS3_WT comparison; (J). Line plot indicating dPSI changes of AS events in 1q amplification and 1q gain comparison.

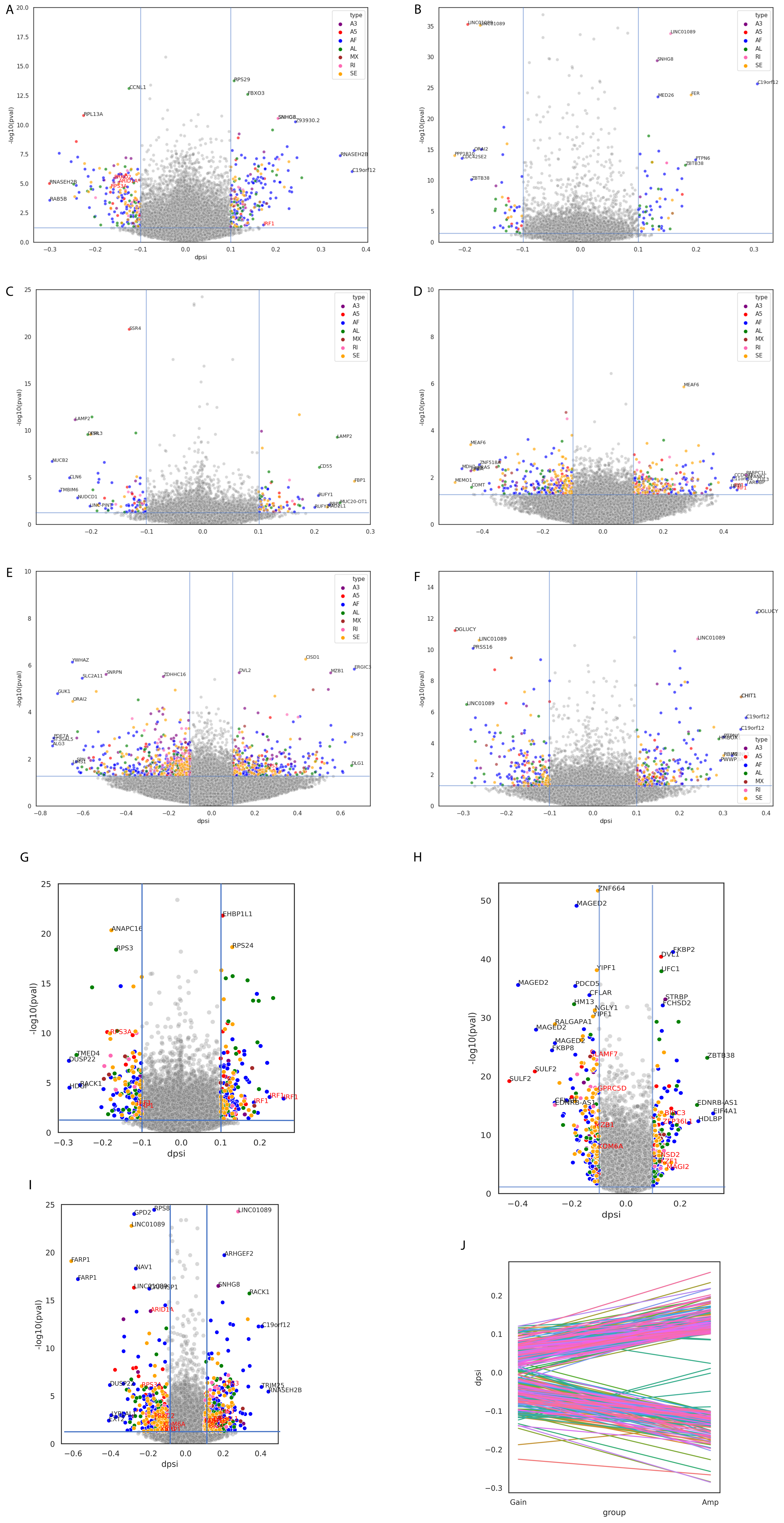

Supplementary Figure 4. Event consistency, Schematic plots of AS events and corresponding transcripts. (A). Venn diagram of events among three translocation comparisons. (B). Venn diagram of events among three DIS3 comparisons. (C). Venn diagram of events among three TENT5C comparisons. (D). Venn diagram of events among three TP53 comparisons. (E). Venn diagram indicating the consistent events among two DIS3-related comparisons and t(14;16) comparisons.
(F). Venn diagram of events between biallelic DIS3 + t(4;14) and biallelic DIS3 - t(4;14), vs DIS3 wild type + no-t(4;14) comparisons.
(G). Schematic plot indicating the different transcript fates due to a SE event in YIPF1.
(H). Schematic plot indicating the domain changes due to an MX event in SLAMF7.

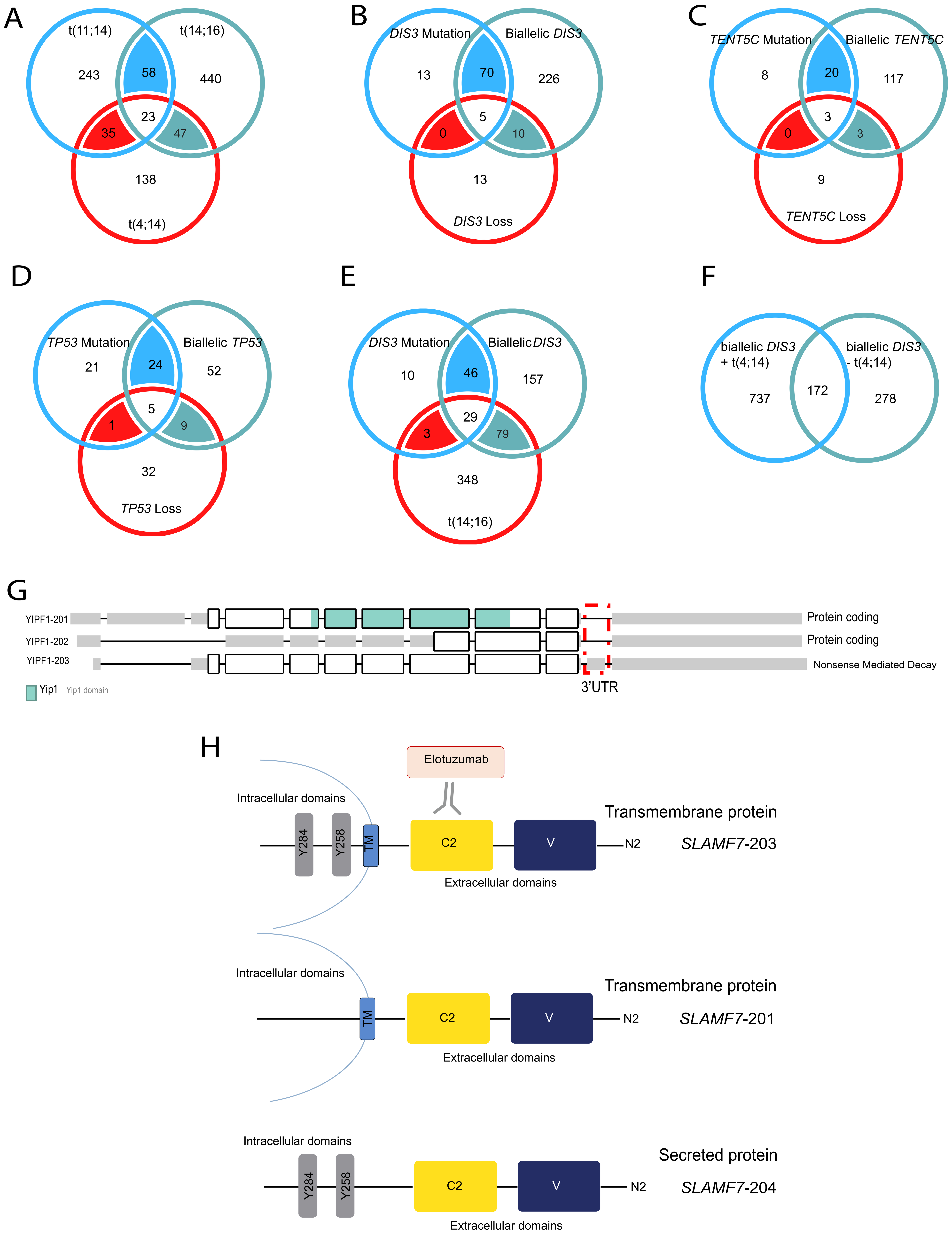

Supplementary Figure 5. Dysregulated Gene Ontology Biological Process (GOBP) from differentially spliced genes in all comparisons from GSEA analysis

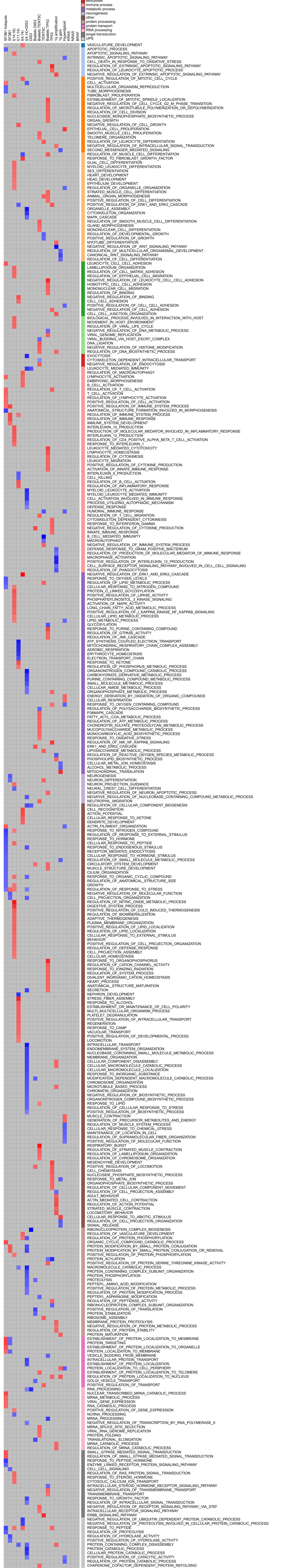

Supplementary Figure 6. of spliceosome genes across comparisons

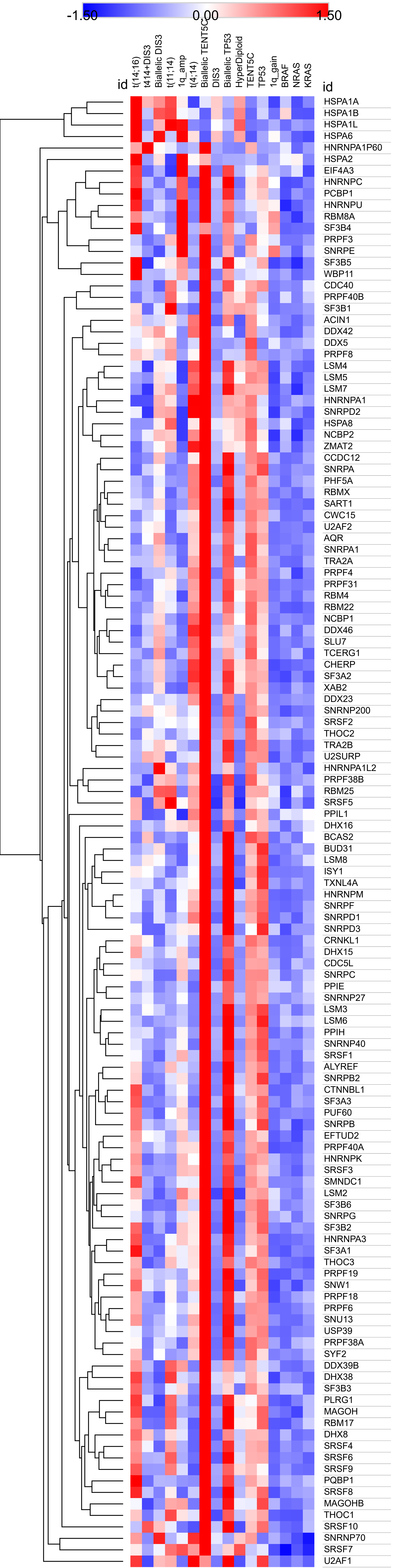

Supplementary Figure 7. Heatmaps of pathway gene expressions across 598 NDMM samples

A. Heatmap of DNA repair pathway signature (1)

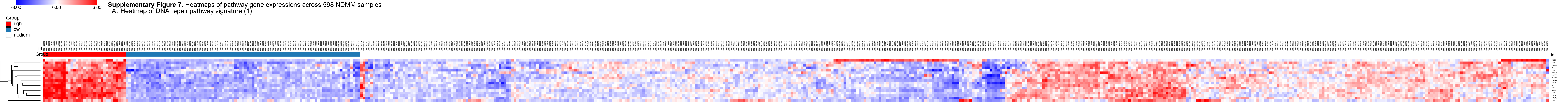

B. Heatmap of KEGG spliceosome pathway

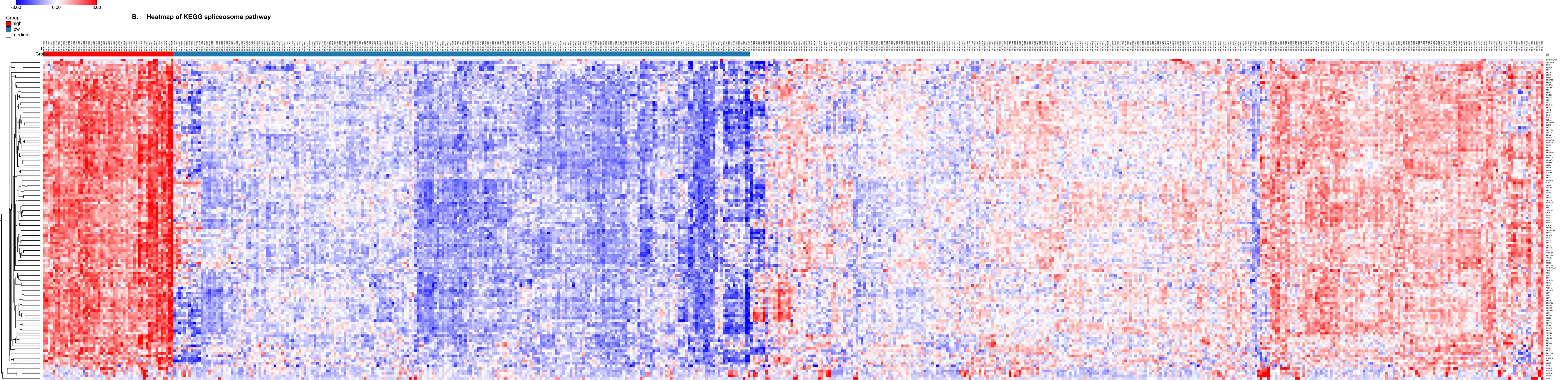

C. Heatmap of KEGG proteasome pathway

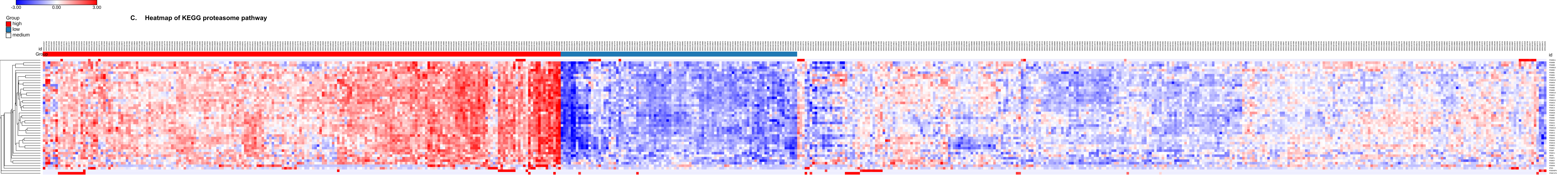

D. Heatmap of KEGG ubiquitin mediated proteolysis pathway

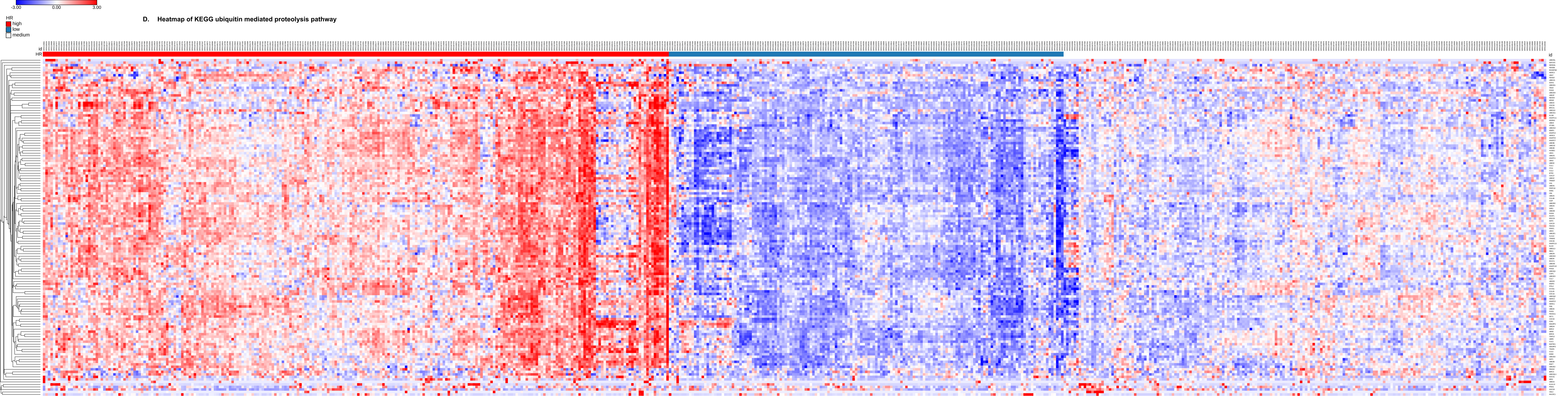

E. Heatmap of KEGG Non-Homologous End Joining pathway

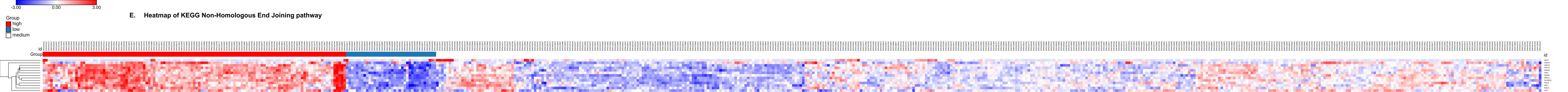

F. Heatmap of KEGG Homologous Recombination pathway

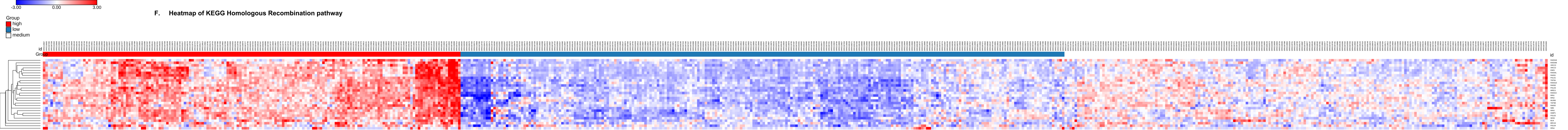

G. Heatmap of Microhomology Mediated end joining pathway. Gene list is obtained from a previous study (2).

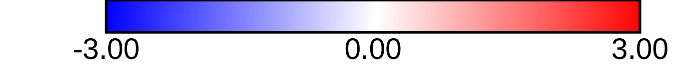

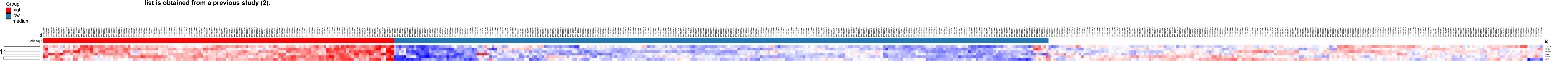

Supplementary Figure 8. Features of NHEJ-high -medium and -low groups. (A). Expression difference of 11 NHEJ genes between -high and -low roup. Stats: Independent T-test

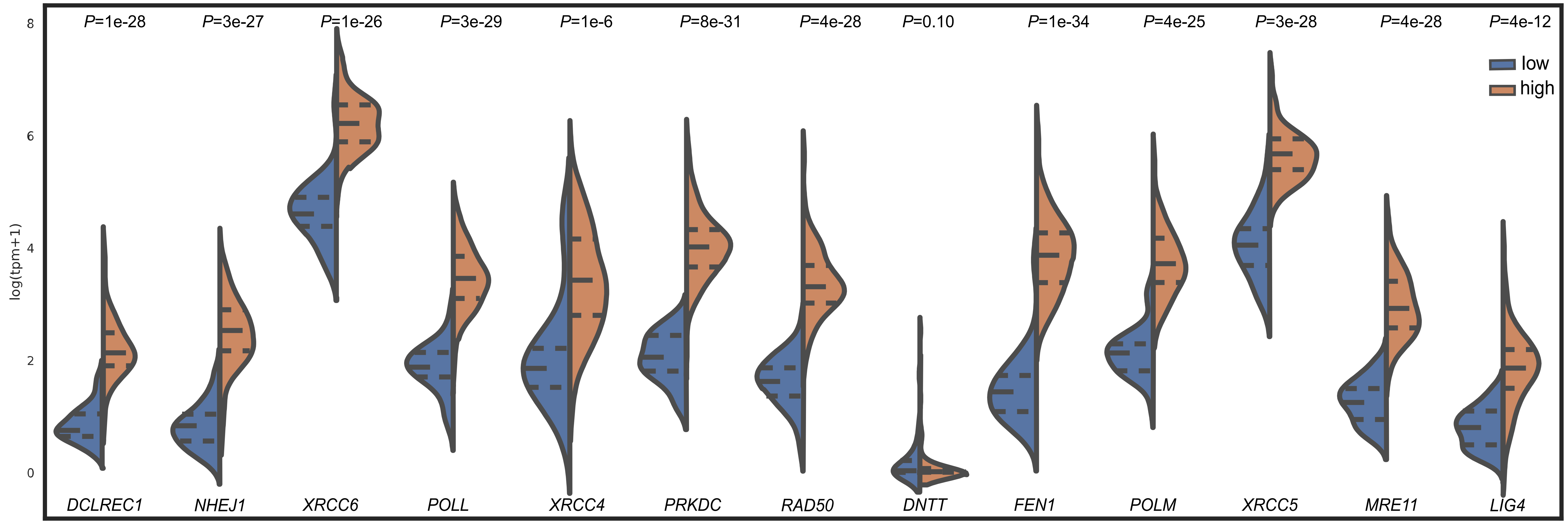

Supplementary Figure 8. Features of NHEJ-high -medium and -low group.
(B). Elevated expression levels of 69 splicing factors in NHEJ-low (green), via-Medium (orange) and -high (blue) group. X-axis: groups. Y- axis: Log2(TPM+1)

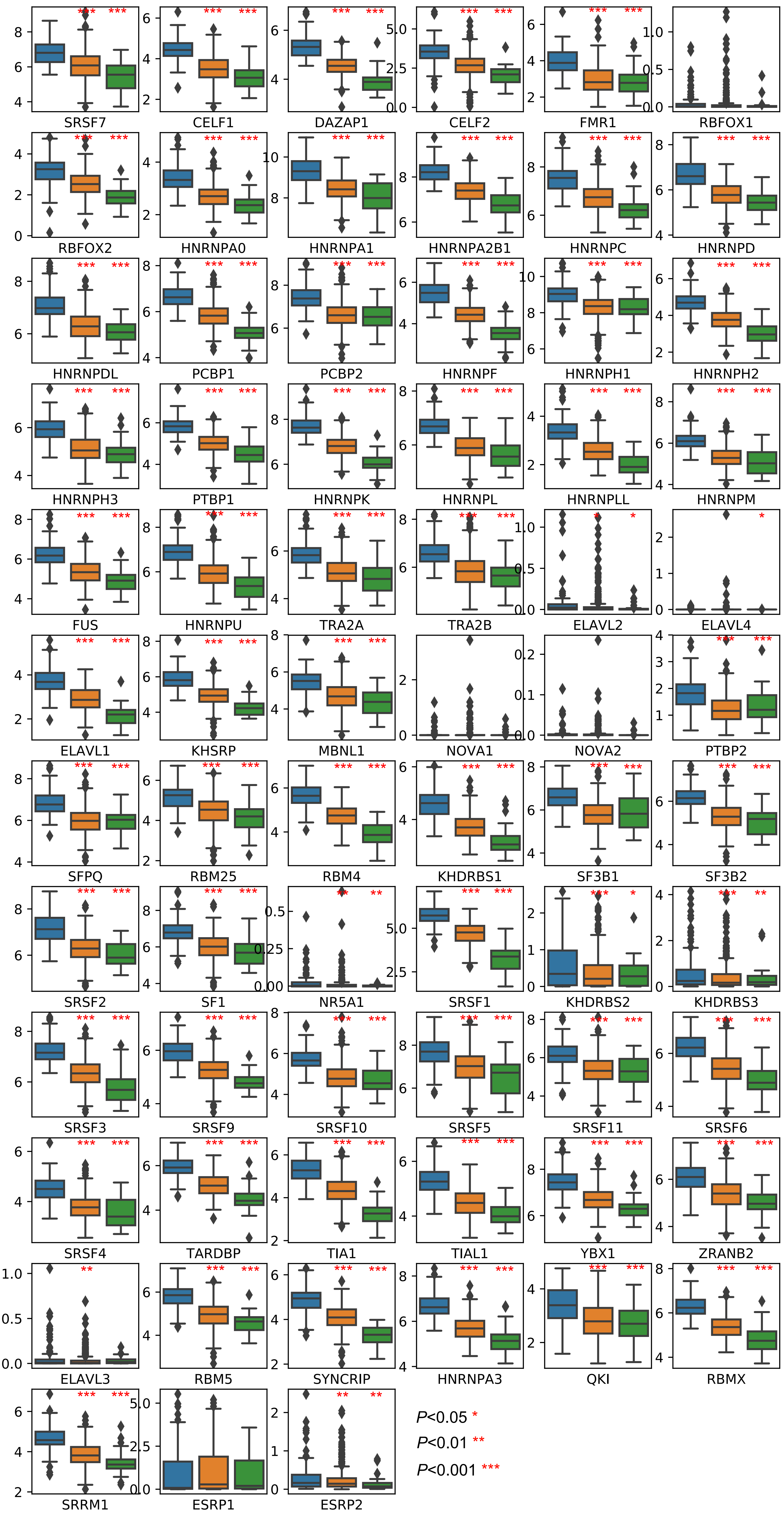

Supplementary Figure 9 . Transcripts involved in RHOQ_AF events and AS markers in prognostic signatures. (A). Expression level of transcripts RHOQ-202 and RHOQ-205. (B). Schematic plot indicating the domain changes and different fate between RHOQ-202 and RHOQ-205 due to an AF event. (C). Sashimi plot of the SE event in ETFA. FA-201 and ETFA-207 in <mean and >mean group defined by mean PSI of (C). (E). Sashimi plot of AF event in TANK. (F). Expression difference of TANK-201 and TANK-202 in <mean and >mean group defined by mean PSI of (E). Statistics: One-sided T-test.

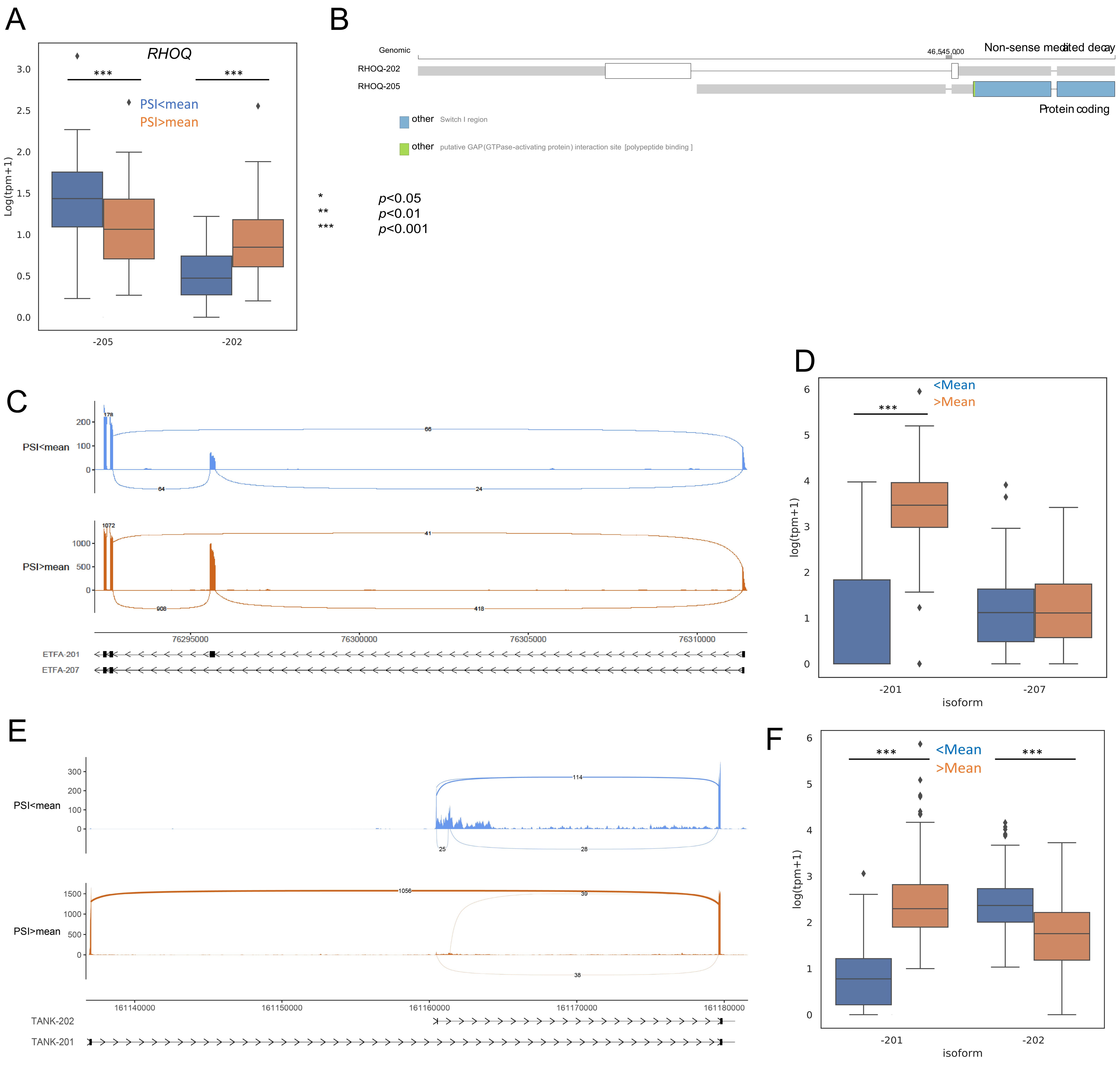

Supplementary Figure 9 (cont.). AS markers in prognostic signatures (G). Sashimi plot indicating the SE event in TSPAN3 (H). Expression levels of TSPAN3-201 and ETFA-203 in <mean and >mean group defined by mean PSI of event (G). (I). Sashimi plot indicating the MX event in PVT1 (J). Expression levels of PVT1-315, PVT1-224 and PVT1-224 in <mean and >mean group defined by the mean PSI of event (I). (K). Sashimi plot indicating the MX event in BUD23 (L). Expression levels of BUD23-205 and BUD23-208 in <mean and >mean group defined by the mean PSI of event (K). Statistics: One-sided T-test.

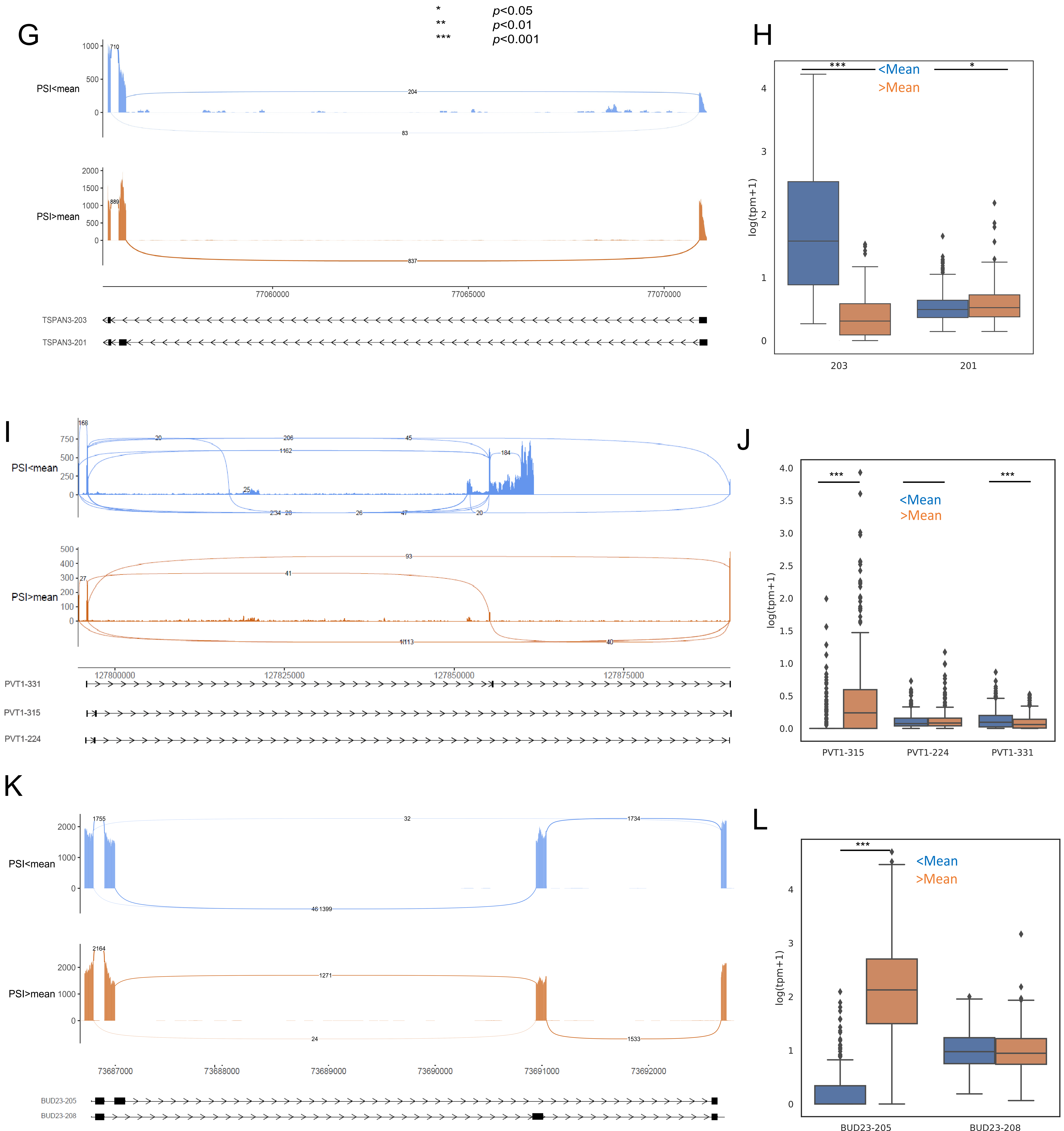

Supplementary Figure 9 (cont.) AS markers in prognostic signatures. (M). 5 AS events associated with poor prognosis

M

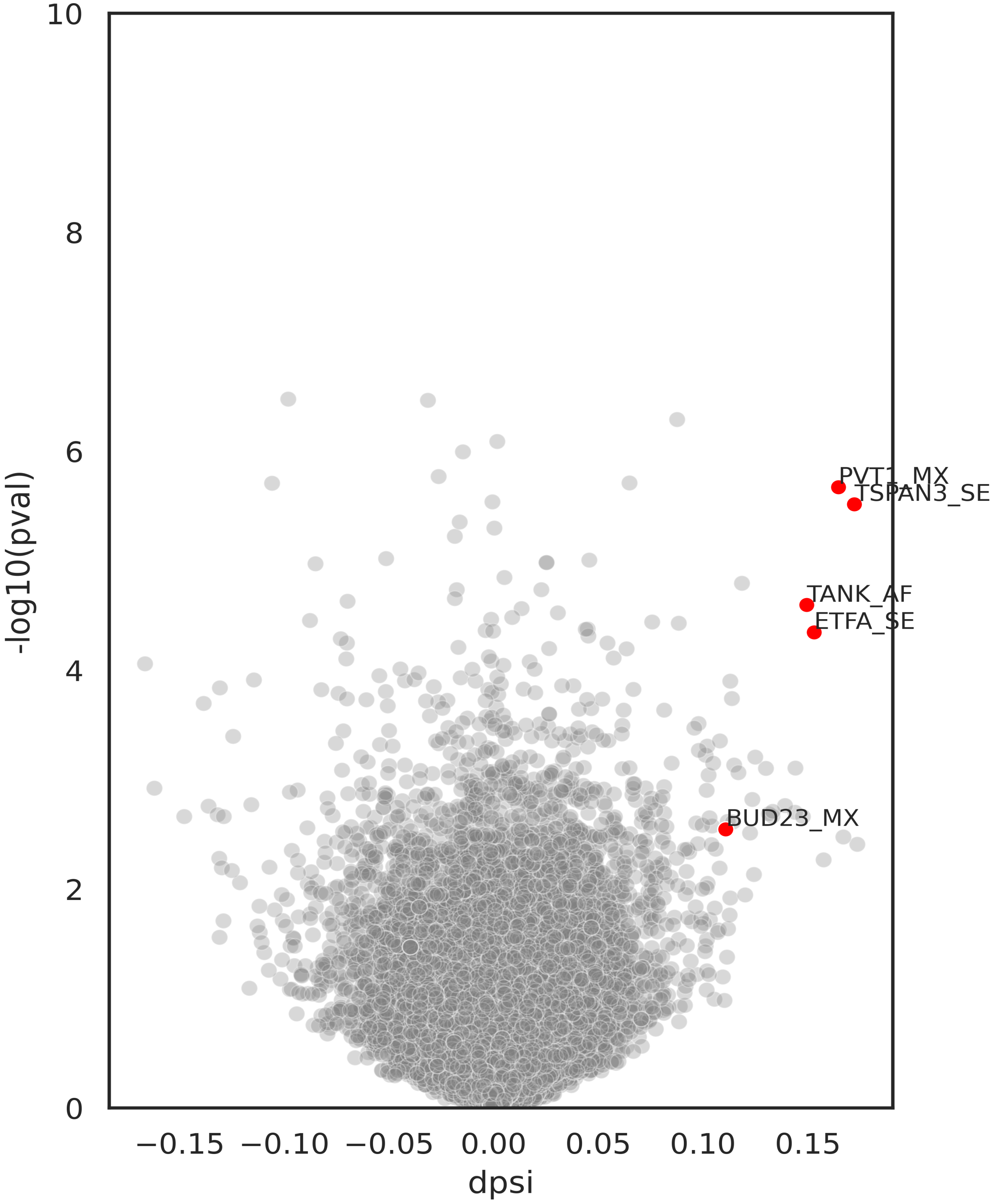

Supplementary Figure 10. Correlation networks between SDS events and splicing factors of (A): t(14;16) and (B). t(4;14). (C). Schematic plot of transcripts involved in an AL event in ZFAS1. (D). Expression difference of ZFAS1-203 and ZFAS1-210 in t(14;16) and non-translocated samples.

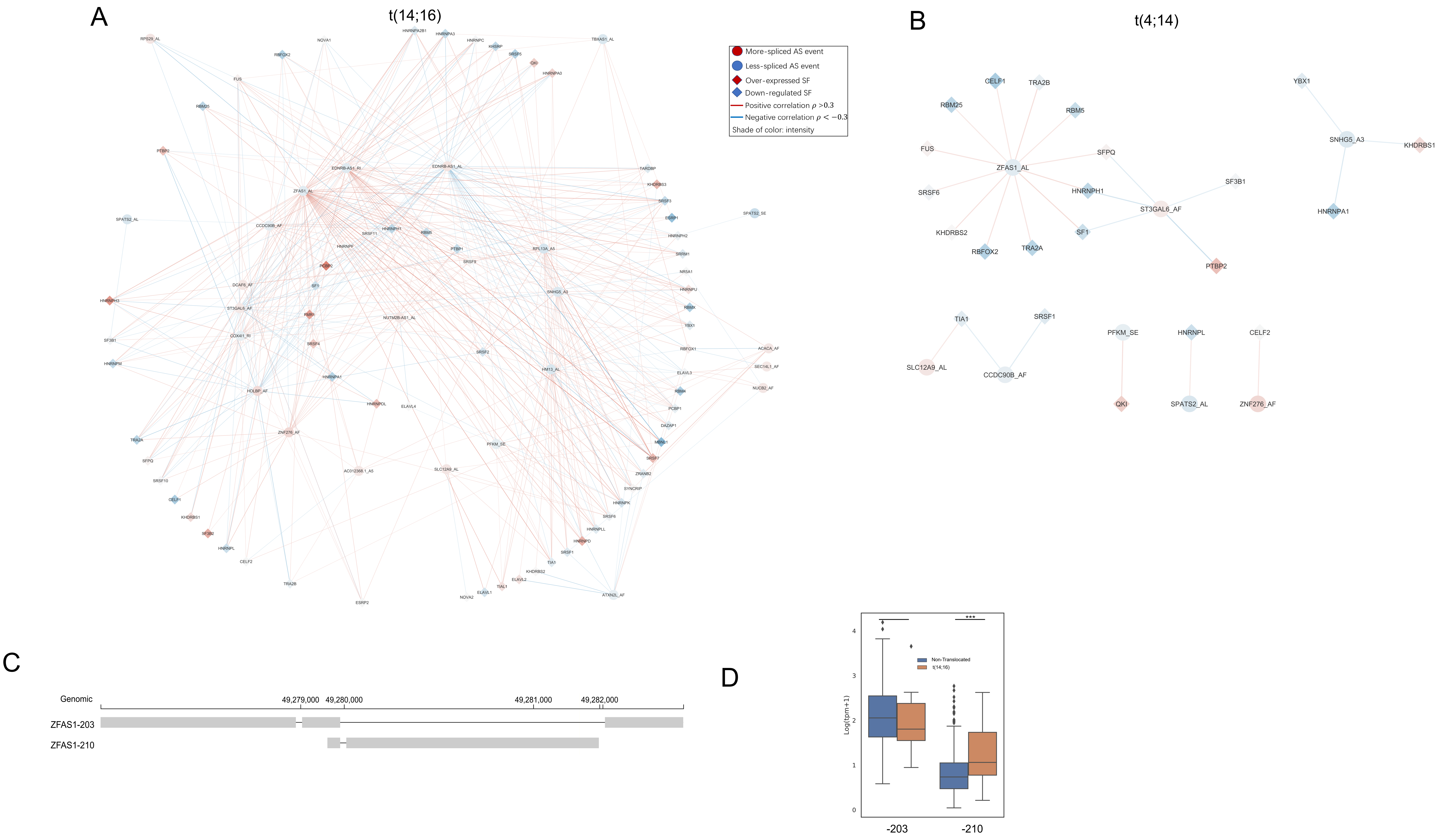

Supplementary References

1. Sharma S, Javadekar SM, Pandey M, Srivastava M, Kumari R, Raghavan SC. Homology and enzymatic requirements of microhomology-dependent alternative end joining. Cell Death Dis. 2015;6(3):e1697-e.
2. Kassambara A, Gourzones-Dmitriev C, Sahota S, Rème T, Moreaux J, Goldschmidt H, et al. A DNA repair pathway score predicts survival in human multiple myeloma: the potential for therapeutic strategy. Oncotarget. 2014;5(9):2487.
3. Rouillard AD, Gundersen GW, Fernandez NF, Wang Z, Monteiro CD, McDermott MG, et al. The harmonizome: a collection of processed datasets gathered to serve and mine knowledge about genes and proteins. Database. 2016;2016.
4. Cunningham F, Allen JE, Allen J, Alvarez-Jarreta J, Amode MR, Armean IM, et al. Ensembl 2022. Nucleic Acids Res. 2022;50(D1):D988-D95.
5. Lamy L, Ngo VN, Emre NCT, Shaffer Iii AL, Yang Y, Tian E, et al. Control of autophagic cell death by caspase-10 in multiple myeloma. Cancer Cell. 2013;23(4):435-49.
6. Zhou W, Zhao L, Yuan H, Xu L, Tan W, Song Y, et al. A new small cell lung cancer biomarker identified by Cell-SELEX generated aptamers. Exp Cell Res. 2019;382(2):111478.
7. Kwon HY, Bajaj J, Ito T, Blevins A, Konuma T, Weeks J, et al. Tetraspanin 3 is required for the development and propagation of acute myelogenous leukemia. Cell stem cell. 2015;17(2):152-64.
8. Molyneux SD, Waterhouse PD, Shelton D, Shao YW, Watling CM, Tang Q-L, et al. Human somatic cell mutagenesis creates genetically tractable sarcomas. Nat Genet. 2014;46(9):964-72.
